# Biomarkers of endothelial activation and inflammation in dogs with organ dysfunction secondary to sepsis

**DOI:** 10.3389/fvets.2023.1127099

**Published:** 2023-07-13

**Authors:** Sarah Gaudette, Lisa Smart, Andrew P. Woodward, Claire R. Sharp, Dez Hughes, Simon R. Bailey, Julien R. S. Dandrieux, Leilani Santos, Manuel Boller

**Affiliations:** ^1^Melbourne Veterinary School, Faculty of Veterinary and Agricultural Sciences, University of Melbourne, Melbourne, VIC, Australia; ^2^School of Veterinary Medicine, Murdoch University, Perth, WA, Australia; ^3^Small Animal Specialist Hospital, Tuggerah, NSW, Australia; ^4^Center for Terrestrial Ecosystem Science and Sustainability, Harry Butler Institute, Murdoch University, Murdoch, WA, Australia; ^5^VCA Canada Central Victoria Veterinary Hospital, Victoria, BC, Canada

**Keywords:** endothelium, sepsis, glycocalyx, inflammation, canine, organ dysfunction

## Abstract

**Introduction:**

Alteration in endothelial function during sepsis is thought to play a key role in the progression of organ failure. We herein compared plasma concentrations of endothelial activation biomarkers vascular endothelial growth factor (VEGF), hyaluronan (HA), plasminogen activator inhibitor-1 (PAI-1) and von Willebrand factor (vWF), as well as inflammatory mediator concentrations (IL-6, IL-8, IL-10, C-reactive protein and monocyte chemoattractant protein-1) in dogs with sepsis to healthy dogs.

**Methods:**

This study was a multicenter observational clinical trial conducted at two university teaching hospitals from February 2016 until July 2017. The study included 18 client-owned dogs hospitalized with sepsis and at least one distant organ dysfunction, as well as 20 healthy dogs. Plasma biomarker concentrations were measured using ELISA. Severity of illness in dogs with sepsis was calculated using the 5-variable acute physiologic and laboratory evaluation (APPLE_FAST_) score. Biomarker concentrations were compared between septic and healthy dogs using linear models.

**Results:**

Septic peritonitis was the most frequent source of sepsis (11/18; 61%), followed by pneumonia (4/18; 22%). Ten dogs (56%) had only 1 organ dysfunction, whereas 3 dogs (17%) had 2, 3 (17%) had 3, 1 (6%) had 4 and 1 (6%) had 5 organ dysfunctions. The median APPLE_FAST_ score in the septic dogs was 28.5 (Q1-Q3, 24–31). Mean plasma concentrations of all endothelial and inflammatory biomarkers, except vWF, were higher in the sepsis cohort than in controls. The mean endothelial biomarker concentrations in the septic cohort ranged from ~2.7-fold higher for HA (difference in means; 118.2 ng/mL, 95% credible limit; 44.5–221.7) to ~150-fold for VEGF (difference in means; 76.6 pg./mL, 95% credible limit; 33.0–143.4), compared to the healthy cohort. Fifteen dogs with sepsis (83%) died; 7 (46%) were euthanized and 8 (53%) died during hospitalization.

**Conclusion:**

Dogs with naturally occurring sepsis and organ dysfunction had higher mean concentrations of biomarkers of endothelial activation and inflammation compared to healthy dogs, broadening our understanding of the pathophysiology of sepsis secondary to endothelial dysfunction.

## 1. Introduction

The syndrome of sepsis in people and animals represents a wide spectrum of illness severity, with a complex pathophysiology that can lead to multiple organ dysfunction. In people, the risk of death increases proportionately with number and severity of organ dysfunctions (1). In dogs, the reported mortality rate associated with sepsis also depends on illness severity, with at least 3 organ systems failing associated with approximately 70–80% non-survival (2, 3). The progression to organ dysfunction, at sites distant to the source of infection, is proposed to be due to a dysregulated host response, whereby the immune, coagulation and endothelial systems lose homeostasis (4).

Alteration in endothelial function and microvascular perfusion during sepsis is thought to play a key role in the progression of organ failure (5, 6). Under systemic inflammatory conditions such as sepsis, the endothelium becomes activated, developing a proinflammatory, procoagulant, hyperpermeable and vasodilatory state (7). At a molecular level, activation of the endothelium involves shedding of its surface layer, the endothelial glycocalyx, upregulation of adhesion molecules to facilitate platelet and leukocyte interaction, and release of a range of molecules involved in angiopoiesis, vascular permeability and coagulation (8). Many of the molecules involved in this process can be measured in the circulation as biomarkers of endothelial activation (9).

The endothelium has been increasingly recognized as a key player in the pathophysiology of organ dysfunction secondary to sepsis. Research investigating the use of soluble biomarkers to allow for the objective assessment of endothelial activation/dysfunction is being pursued. Numerous studies in septic people have demonstrated increased serum or plasma endothelial biomarker concentrations, and many of these biomarkers have shown prognostic utility in predicting illness severity and mortality (10–14). Studies investigating biomarkers of inflammation, endothelial activation and glycocalyx shedding in dogs with sepsis are limited. Characterizing elevations of these biomarkers in septic dogs with organ dysfunction may enhance our understanding of the role of the endothelium in sepsis pathophysiology, as well as identify prognostic potential.

The objective of this preliminary study was to compare plasma endothelial activation biomarker concentrations in dogs with sepsis to healthy dogs. We hypothesized that septic dogs would have an increase in four biomarkers of endothelial activation: vascular endothelial growth factor (VEGF), a mediator of vascular permeability; hyaluronan (HA), a component of the endothelial glycocalyx; plasminogen activator inhibitor 1 (PAI-1), a regulator of fibrinolysis; and von Willebrand factor (vWF), a key hemostatic factor. Given the relationship between endothelial activation and inflammation, we also compared several biomarkers of inflammation between groups; C-reactive protein (CRP), interleukin (IL)-6, IL-8, IL-10 and monocyte chemoattractant protein-1 (MCP-1).

## 2. Materials and methods

### 2.1. Study design

This was a multicenter, prospective, observational study conducted in the intensive care units of two veterinary teaching hospitals between February 2016 and July 2017. Approval from the Animal Ethics Committees of both the University of Melbourne (permit number: 1513658) and Murdoch University (permit number: R2794/15) were received before study commencement. Informed owner consent was obtained for client-owned dogs treated for sepsis at either participating institution, as well as healthy staff- or student-owned dogs that served as a control group.

Dogs were classified as having sepsis according to three criteria, which align with the 2012 Surviving Sepsis Campaign definition of ‘severe sepsis’ (15). First, at least two out of four of the following markers of the systemic inflammatory response syndrome (SIRS): hypothermia or hyperthermia (<37.8°C or > 39.4°C), tachycardia (heart rate > 140/min), tachypnea (respiratory rate > 20/min), leukopenia (<6.0 ×10^9 /L) or leukocytosis (>16.0×10^9 /L) or an increase in band neutrophils (>3% of white blood cell count). Second, the presence of bacterial infection defined as either the clinical suspicion of infection (as deemed by the treating clinician and criticalist reviewing the medical records) or confirmation of infection based on cytology or culture. Third, the presence of at least one distant organ dysfunction: renal, respiratory, cardiovascular, coagulation, or hepatic (2). Renal dysfunction was defined as a urine output <0.5 mL/kg/h (if a closed urinary collection system was in place) or serum creatinine concentration > 1.8 mg/dL (>159 umol/L). Respiratory dysfunction was defined as the need for mechanical ventilation, supplemental oxygen to maintain the SpO_2_ > 95%, or a PaO_2_/FiO_2_ < 300. Cardiovascular dysfunction was defined as administration of a vasopressor drug for hypotension within 24 h of sample collection or a single episode of fluid-unresponsive hypotension, defined as a Doppler blood pressure < 90 mmHg after receiving at least 20 mL/kg of a crystalloid fluid bolus. Coagulation system dysfunction was defined as the presence of thrombocytopenia (<100 ×10^9 platelets/L), prothrombin (PT) and/or activated partial thromboplastin (aPTT) time prolonged more than 25% above the upper reference limit, and/or viscoelastic test abnormalities consistent with hypocoagulability (ROTEM**®** Delta, Werfen, or TEG 5000**®**, Haemonetics). Hepatic dysfunction was defined by an increase in serum bilirubin concentration of at least two times the upper reference interval limit. Dogs were excluded from the study if they were < 5 kg, <6 months of age or treated with corticosteroids in the 7 days preceding study enrolment. Dogs were also excluded if blood sampling could not safely occur, defined as a hematocrit or packed cell volume < 20%, platelet count <50×10^9 platelets/L, and PT or aPTT above the measurable limit, in combination with lack of a sampling catheter. Dogs with a known history of concurrent chronic disease were not excluded; however, this information was recorded.

Healthy dogs were volunteered staff- or student-owned dogs that were considered systemically healthy based on history, physical examination, and routine hematological and biochemical parameters within the reference intervals. Dogs were excluded if they were receiving any medications other than routine flea and worm prophylaxis.

### 2.2. Clinical data collection

Data acquisition was planned to occur for dogs with sepsis as soon as the inclusion criteria were met, and research staff were alerted. Variables collected included age, weight, sex, de-sexing status, comorbidities, and current medications. Clinical variables collected at time of enrolment included heart rate, respiratory rate, rectal temperature, mean arterial or Doppler systolic blood pressure, mentation, complete blood count and plasma biochemistry data, and source of sepsis. The acute patient physiological and laboratory evaluation fast score (APPLE_FAST_) was calculated using variables (glucose, albumin, lactate, platelet count and mentation score) collected at the time of enrolment (16). Survival to discharge was recorded for all dogs. In the event of euthanasia, primary clinicians were asked to characterize their perception for the motivation for the decision as futility or financial, using a sliding scale for each of the two factors. On that scale, “1” indicated no influence on the decision and “10” the only reason for the decision.

Clinical data was initially collected on a case report form and then transcribed into an electronic, internet-based database (REDCap) specific to the study, allowing central administration of data from both enrolment centers (17). The electronic database was backed up daily.

### 2.3. Sample collection and biomarker measurement

Venous blood (10 mL) was collected at the time of enrolment and placed into one EDTA and one sodium citrate vacuum collection tube (Vacutainer tubes, Beckton Dickinson, Vacutainer Systems, Franklin Lakes, NJ). Samples were collected prior to administration of a fluid bolus or at least 30 min after the completion of a fluid bolus. The samples were centrifuged at 1,500xg for 15 min at 5°C within 1 h of sample collection. The plasma was removed and separated into 500 μL aliquots in individual screw top microcentrifuge tubes (2241-SO, Scientific Specialties Incorporated, Lodi, CA, United States) and stored at −80°C until batch analysis was performed. Biomarker measurement was conducted at a single site. All samples collected at the other institution were transported together *via* overnight courier using a validated dry ice packaging system (Orca S, Intelsius, VIC, Australia) at completion of enrolment. Upon arrival the still frozen samples were transferred immediately into an ultracool (−80°C) freezer until later batch analysis. Samples underwent no more than 2 freeze–thaw cycles before biomarker measurement.

Biomarker measurements, using commercial canine quantitative sandwich ELISA kits validated for use in the dog, were performed as per manufacturer instructions. This included VEGF and HA (both Quantikine ELISA kits, R&D Systems, Minneapolis, MN, United States), PAI-1 (Canine PAI-1 total antigen assay ELISA kit, Molecular Innovations, Novi, MI, United States) and C-reactive protein (CRP) (TE1024, TECOmedical Group, Sissach, Switzerland). Other inflammatory biomarkers were measured using a magnetic bead-array multiplex panel also validated for use in the dog (Merck Millipore, MA, United States) and included interleukin IL-6, IL-8, IL-10 and MCP-1. Plasma canine-specific vWF antigen concentration was determined using a sandwich ELISA developed and validated for vWF analysis in dogs (18, 19). EDTA plasma was used for all measurements except vWF antigen, which used citrated plasma. A facemask was worn during measurement of VEGF and HA to prevent contamination from saliva. Each sample was analyzed in duplicate and the mean carried forward. For biomarker concentrations that fell below or above the detection limit of the respective ELISA or multiplex assay, the concentration was recorded as either the lower or upper limit of detection, respectively.

### 2.4. Statistical analysis

Data analyzes were conducted in R (20). Biomarker concentrations and demographic variables were summarized using the median and quartiles. Due to the abundance of left- and right-censored observations for many of the responses (those that were either below or above the reporting limits of the analytic assay, respectively) (Table 1), an analysis strategy was selected that incorporated the incompleteness of measurement of those observations. To determine the effect of the sepsis state on the responses compared to controls, the selected model for each response was a Bayesian formulation of the *t*-test which accepted censoring of the responses (21, 22). This was a linear model of the form:

$$\log_{e}\left(y\right)\sim \beta_{0}+\beta_{1}\left(\text{status}\right)+N\left(0,\sigma_{\text{res}}\right)$$

where status is the disease state (sepsis or control), and y is the response variable, which may be left-censored, right-censored, or both, which is conditionally normal with residual standard deviation $\sigma_{\text{res}}$ (as in the typical *t*-test). This model was implemented using the ‘brms’ package in R (23). Priors for $\beta_{1}$ were weakly-informative, N(0,σ), with σ selected after examining histograms of the log-transformed responses. Goodness-of-fit of the models was assessed by residual analysis, but with consideration of the censoring limits. Estimates of the effect of status, and of the predicted mean concentrations in the control and sepsis conditions, were summarized using the posterior mean 95% credible intervals.

**Table 1:** Plasma concentrations of biomarkers in dogs with sepsis and in healthy control animals.

Biomarker	Control (*n* = 20)			Sepsis (*n* = 18)		
	Median (range)	Number <LLOD	Number >ULOD	Median (range)	Number <LLOD	Number >ULOD
Endothelial						
VEGF (pg/mL)	39.1 (N/A)	20	0	50.4 (39.1–659.5)	8	0
HA (ng/mL)	76.1 (26.2–147.4)	0	0	244.9 (21.3–251.8)	0	9
vWF (ng/mL)	118 (56–198)	0	0	110 (24–491)	0	0
PAI-1 (ng/mL)	25.1 (4.8–62.3)	0	0	87.5 (30.3–1028.6)	0	1
Inflammation						
CRP (μg/mL)	19.1 (19.1–87.3)	17	0	199.9 (80.2–392.0)	0	0
IL-6 (pg/mL)*	51.2 (51.2–11,541)	12	0	3092.5 (206.4–39,852)	0	0
IL-8 (pg/mL)*	483.0 (94.9–1776)	2	0	3554.0 (306.0–118,918)	0	0
IL-10 (pg/mL)*	73.0 (73.0–732.7)	14	0	198.8 (73.0–1938)	6	0
MCP-1 (pg/mL)*	788.4 (132.4–1,285)	3	0	6974.4 (1939–203,884)	0	1

*Two samples missing from the control group due to inadequate sample volume. The table includes the number of samples that were censored due to measuring either below the lower limit of detection (LLOD) or above the upper limit of detection (ULOD).

## 3. Results

### 3.1. Animal characteristics

Twenty healthy dogs were included in the control group, as planned. Twenty dogs met the inclusion criteria for sepsis, however two were later excluded on review due to alternative diagnosis made (hypoadrenocorticism) or lacking organ dysfunction. Both healthy dogs and septic dogs represented a variety of breeds (see Supplementary Table S1). Median age (Q1 – Q3) of the healthy dogs was 5.5 years (0.7–11) and of the septic dogs was 8.4 years (3.2–15). The healthy dogs included 7 castrated males, 2 sexually intact females and 11 spayed females. The septic dogs included 3 sexually intact males, 7 castrated males, 2 intact females and 6 spayed females. The median weight of the healthy dogs was 24.3 kg (6.1–37.0) and of the septic dogs was 28.0 kg (7.1–45.2).

Comorbidities present in the sepsis group were idiopathic epilepsy (2), diabetes mellitus (2), osteoarthritis (1), polyradiculoneuritis (1), elapid snake envenomation (1), nasal aspergillosis (1), ophthalmic disease (1) and recent trauma (1). One dog had a medication history of phenobarbitone and potassium bromide for idiopathic epilepsy, two dogs were being administered non-steroidal anti-inflammatory medication and two dogs were receiving insulin therapy prior to hospitalization. Antimicrobial drugs had been administered to 16 dogs prior to sample collection and eight dogs had been treated with sedatives within 24 h of enrolment.

Septic peritonitis was the most frequent source of sepsis (11/18; 61%), followed by pneumonia (4/18; 22%), hepatitis (2/18; 11%), and necrotizing mastitis (1/18; 6%). Infection was suspected in 6/18 (33%) and confirmed in 12/18 (67%) dogs *via* body fluid analysis of either peritoneal fluid (10/12), bronchoalveolar lavage fluid (1/12) or mammary gland abscess fluid (1/12). This consisted of either cytological identification of intracellular organisms (6/12) or positive fluid culture (6/12). Over half of the septic dogs (10/18) had only 1 organ dysfunction, with the remaining dogs having either 2 (3/18), 3 (3/18), 4 (1/18) or 5 (1/18) organ dysfunctions. Coagulation system dysfunction was the most frequent type of organ dysfunction (10/18; 56%), followed by cardiovascular (7/18; 39%), respiratory (7/18; 39%), renal (5/18; 28%) and hepatic (2/18; 11%) dysfunction.

### 3.2. Biomarkers concentrations

**Figure 1:**
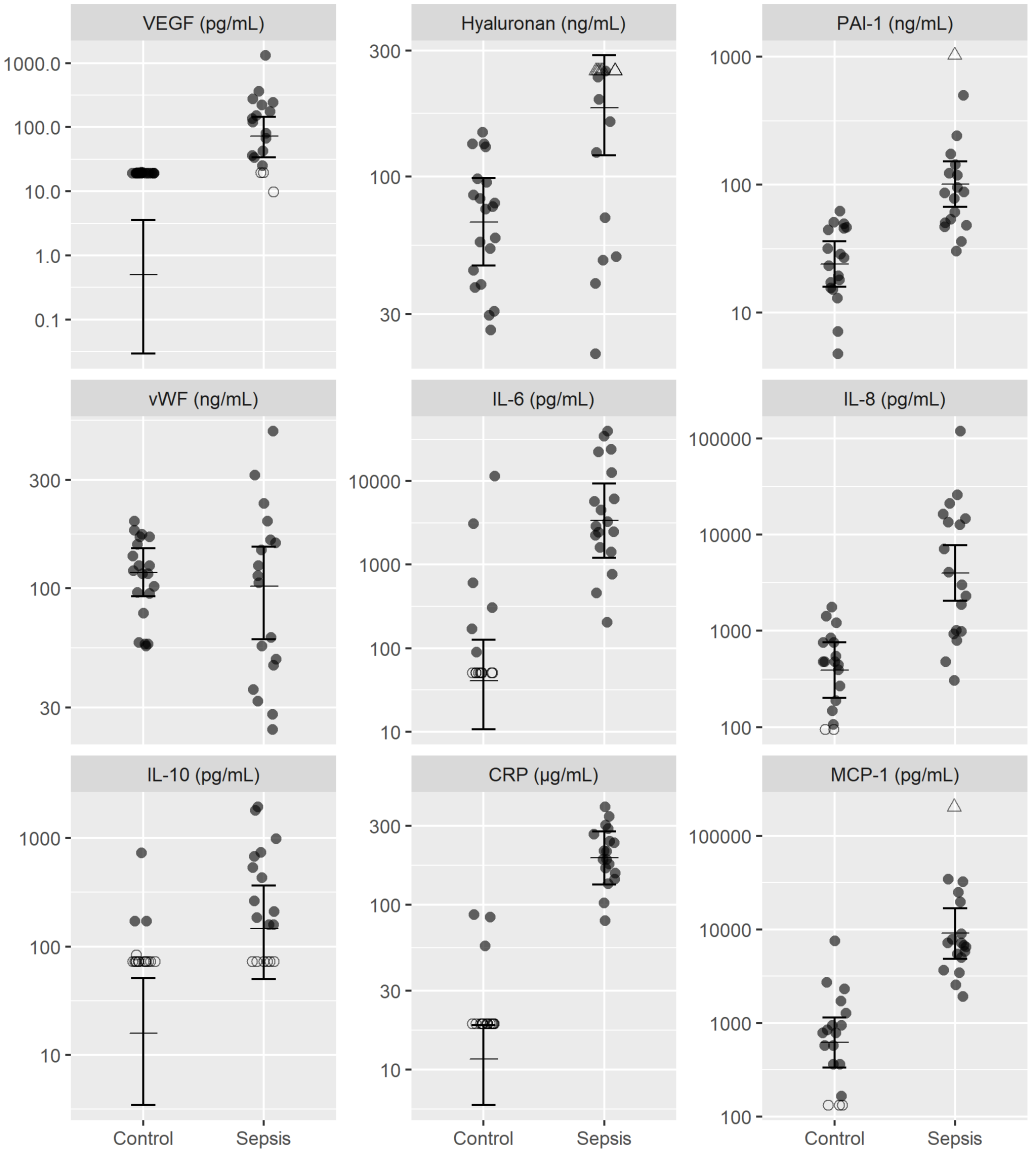
Observed and estimated concentrations of biomarkers of endothelial injury and inflammatory mediators, in septic and control dogs. For each group, the central horizontal line is the estimated (posterior) mean concentration, and bars are 95% credible intervals for the mean concentration, drawn from the final statistical model. All biomarker concentrations are presented on logarithmic (log_10_) scale. Open triangles (△), right-censored observations; open circles (〇), left-censored observations. Note that for left-censored observations, any value below the censoring limit is equally plausible. CRP, C-reactive protein; MCP-1, monocyte chemoattractant protein-1; PAI-1, platelet activator inhibitor 1; VEGF, vascular endothelial growth factor; vWF, von Willebrand factor.

Dogs with sepsis had higher plasma VEGF, HA, and PAI-1 concentrations, compared to healthy dogs (Figure 1; Table 2). There was little apparent difference in mean plasma vWF concentration between groups, but some evidence that vWF was more variable in dogs with sepsis. Dogs with sepsis also had significantly higher plasma IL-6, IL-8, IL-10, CRP and MCP-1 concentrations, compared to healthy dogs (Figure 1; Table 2).

**Table 2:** Inferential estimates of plasma concentrations of markers of endothelial activation and inflammation.

Biomarker	Control			Sepsis			Estimated Effect		
	Mean	Credible Limits		Mean	Credible Limits		Difference in means (Sepsis–Control)	Credible Limits	
		2.5%	97.5%		2.5%	97.5%		2.5%	97.5%
Endothelial									
VEGF (pg/mL)	0.499	0.00293	3.56	72.6	33.8	144.4	76.6	33.0	143.4
HA (ng/mL)	67.2	46.0	98.5	182.1	120.0	288.0	118.2	44.5	221.7
vWF (ng/mL)	117.6	92.4	149.7	102.1	59.7	152.8	−13.6	−67.3	41.7
PAI-1 (ng/mL)	23.9	15.8	36.0	101.4	67.3	152.9	79.3	41.0	129.6
Inflammatory									
CRP (μg/mL)	11.6	6.08	18.6	193.3	132.4	279.0	184.7	120.7	268.1
IL-6 (pg/mL)	40.7	10.6	126.5	3,390	1,199	9,363	3,884	1,198	9,362
IL-8 (pg/mL)	390.6	200.7	764.3	3,991	2059	7,752	3,808	1,616	7,348
IL-10 (pg/mL)	15.9	3.45	50.9	145.8	49.9	363.7	144.7	32.6	341.9
MCP-1 (pg/mL)	624.2	332.9	1,140	9,108	4,845	16,910	8,916	4,163	16,240

Means are estimated means for ‘Control’ and ‘Sepsis’ that are predicted from the final models with their credible intervals. The differences in means are in linear scale and determined from samples of the posterior distribution (after transformation from the log_e_-model). A positive difference in means indicates higher concentration in the sepsis cohort. The difference in means, produced from the final statistical models, provides the outcomes of the statistical analysis. CRP, C-reactive protein; HA, hyaluronic acid; MCP-1, monocyte chemoattractant protein-1; PAI-1, platelet activator inhibitor-1; VEGF, vascular endothelial growth factor; vWF, von Willebrand factor.

### 3.3. Clinical outcomes for dogs with sepsis

The median APPLE_FAST_ score in the septic dogs was 28.5 (Q1-Q3, 24-31). Fifteen dogs were non-survivors (83%), with the remaining 3 dogs (16%) surviving to discharge. Of non-survivors, eight dogs (53%) died naturally and 7 dogs (47%) were euthanized. Perceived reasons for euthanasia, using a sliding scale of 1 to 10, were skewed toward futility rather than financial reasons, with a median futility score of 8 (5-9) versus a median financial score of 3 (1-5). Due to the low number of dogs surviving to discharge, there was inadequate sample size to explore associations between biomarker concentrations and survival.

## 4. Discussion

This study found that dogs with organ dysfunction secondary to sepsis had higher concentrations of VEGF, HA, and PAI-1 compared to healthy control dogs. These findings suggest that endothelial activation occurs in dogs with naturally occurring sepsis and organ dysfunction, which is similar to results found in people with sepsis (24–26). All three endothelial biomarkers are important mediators in systemic inflammation and their increase in dogs with sepsis may explain some of the pathophysiological sequelae observed in clinical patients.

Vascular endothelial growth factor is released from endothelial cells, leucocytes and platelets in response to a range of agonists, including inflammatory cytokines and bacterial elements (27–29). Its actions on the endothelium include increased vascular permeability, vasodilation, and amplification of inflammation and coagulation (30–32). These alterations of the endothelium can lead to interstitial edema, compromised tissue perfusion, and subsequent organ dysfunction. Plasma VEGF concentrations are significantly increased in people with sepsis, compared to healthy volunteers (11, 33, 34). High plasma VEGF concentrations have also been associated with worse clinical outcome in people, such as organ failure and mortality (33), though some studies have conversely reported a negative association between these variables (34, 35).

Two studies have measured plasma VEGF concentration in dogs with SIRS, either due to sepsis or other causes. The first study measured daily plasma VEGF concentration in 28 dogs with either sepsis or non-septic SIRS, and found that 18 of these dogs had a quantifiable concentration of VEGF at one or more time points (36). Although no association was identified between VEGF concentration and clinical evidence of edema, used as a surrogate marker of increased vascular permeability, dogs with very high concentrations (≥ 70 pg./mL; *n* = 3) were more likely to die. A more recent study in dogs with sepsis (*n* = 25) also found significantly increased plasma VEGF concentration, compared to healthy dogs (*n* = 7) (37). This study also included dogs with non-septic SIRS (*n* = 32), and when all dogs were combined, there was some potential identified for VEGF predicting in-hospital mortality (area under the receiving operating characteristic curve 0.68 (95% confidence interval 0.5–0.8); mortality rate 41%). One further study found elevated concentrations of VEGF in dogs with leptospirosis, compared to healthy controls (38), as well as a positive association between VEGF concentration and both inflammatory markers and mortality. Though associations with mortality could not be explored in our study, all three of these preliminary studies suggest that VEGF may play a role in the morbidity of sepsis in dogs, similar to people.

Our study also identified significantly increased plasma concentrations of HA in dogs with sepsis, a component of the endothelial surface layer. Shedding of this surface layer is one of the first steps of endothelial activation, directly leading to a hyperpermeable, proinflammatory and procoagulant phenotype (8). Hyaluronan released into circulation may also stimulate further inflammation. A number of studies in endotoxemia animal models (39–41) and people with sepsis (42–45) have demonstrated significantly increased serum or plasma HA concentrations, compared to baseline or healthy controls, suggesting shedding of the endothelial surface layer. Increased HA concentration has also been associated with degree of organ failure and mortality in people (44, 45). In veterinary medicine, a case series of 8 dogs with septic peritonitis reported a range of serum HA concentration of 18–1,050 ng/mL (46). Although there was not a healthy control comparison reported in this study, this range is considerably higher than observed in our study, lending evidence to elevated HA concentrations in dogs with sepsis. An association between HA and IL6 concentration was also identified. It is important to note that there are other sources of HA in the body, such as the interstitium, and that clinical factors such as rapid intravenous fluid administration can also increase circulating concentrations (47). Therefore, increases in HA concentration may not solely represent endothelial surface shedding.

In addition to biomarkers of endothelial activation or surface shedding, this study also explored biomarkers of coagulation activation, including PAI-1 and vWF. Activation of the endothelium stimulates production of procoagulant mediators, such as tissue factor and vWF, reduces the presence of anticoagulants on the endothelial surface and inhibits fibrinolysis, such as via production of PAI-1 (5, 6). This effect likely contributes to microthrombi formation and development of organ failure. In human clinical studies, PAI-1 concentrations are positively associated with degree of organ failure and mortality in people with sepsis (12, 48–50). In this study, we found that dogs with sepsis had significantly increased plasma concentrations of PAI-1 compared to healthy dogs. Further, coagulation impairment was the most frequent organ dysfunction, as defined by clinicopathologic evidence of hypocoagulability or thrombocytopenia. Plasminogen activator inhibitor-1 may play an important role in the pathogenesis of coagulation complications. Given that disseminated intravascular coagulation is associated with a high mortality rate in dogs (51, 52), it may be clinically useful to identify a biomarker that can predict this complication. Further research is required to determine if elevated PAI-1 concentration has clinical utility in this manner.

Platelet activation is another important contributor to the procoagulant state in sepsis and is stimulated by a range of agonists released by the activated endothelium, including vWF. Endothelial release of highly thrombogenic ultra-large vWF multimers can be stimulated by inflammatory mediators such as IL-8 and tumor necrosis factor-α (53). In people with sepsis, vWF concentrations are significantly higher than healthy controls, though studies have varied in their ability to detect an association with clinical outcome (35, 54, 55). Data from veterinary studies in this area are limited. An experimental study in dogs found a significant rise in plasma vWF antigen concentration after injection of bacterial lipopolysaccharide (56). A clinical study also identified increased vWF antigen concentrations in dogs with naturally-occurring sepsis (*n* = 14), compared to healthy dogs (*n* = 11), but found no evidence of a difference between survivors (*n* = 7) and non-survivors (*n* = 7), probably due to poor power (57). In our study, we did not identify a difference in plasma vWF concentration between healthy dogs and those dogs with sepsis suggesting an inconsistent increase in vWF concentrations subsequent to sepsis. Clearly, the relevance of the observed lack of difference in vWF concentrations in normal and septic dogs requires further investigation.

The increase in endothelial activation biomarkers found in dogs with sepsis in this study concurred with an increase in all inflammatory cytokine concentrations. This finding is consistent with sepsis in people (12, 58). The relationship between endothelial activation and inflammation is likely bidirectional; endothelial mediators can stimulate inflammation and inflammatory cytokines can activate the endothelium. For example, *in vitro* and *in vivo* endotoxin models have shown that VEGF is important for stimulating IL-6 and MCP-1 production and that inflammatory cytokines play a role for both VEGF and PAI-1 release (11, 30, 58). The degree to which the inflammatory cytokine concentrations were increased in dogs with sepsis in our study is consistent with a high severity of illness, with concentrations being many-fold higher on average than in other studies in dogs (59, 60). This is also consistent with the high mortality rate observed in this study.

This preliminary study has several limitations. Some of the biomarkers had observations outside of the detectable range of the assay. Observations outside the validated performance limits of the analytical assays were common, especially in the control cohort, suggesting a need for more sensitive methods. Though models for censored data facilitate valid inference in the presence of those observations, especially regarding large effects and their uncertainty, for some biomarkers we have learned little regarding their distribution in control animals. Despite this limitation, the effect of sepsis on several markers is clearly large and well-estimated, even for the frequently censored biomarkers. However, the study was not designed to, and the sample size was inadequate to, determine correlations between endothelial and inflammatory mediators, or outcome. Methodological error may have also interfered with results. For example, hemolysis, temperature and storage time can have some effect on vWF concentrations (61–63). This may contribute to the lack of a difference in vWF concentrations between healthy and control dogs, in contrast with a previous study (53). It is plausible that biomarker concentrations were also influenced by comorbidities, such as trauma, and not by sepsis alone. Furthermore, inclusion criteria for organ dysfunction in this study selected for septic dogs with a high severity of illness, which must be considered when using this data to inform future biomarker studies. In addition, this study does not provide any information on the specificity of each biomarker, i.e., how well sepsis can be inferred from a known biomarker value. The logical next step, if some of these biomarkers were going to be selected as candidates for a diagnostic test, would therefore be to assess the predictiveness of the selected set of variables for ‘sepsis’ in comparison to clinically similar ‘non-sepsis’. In addition, the association between increases in these markers and endothelial activation/dysfunction needs to be further substantiated.

In conclusion, this study found that endothelial biomarkers, VEGF, HA and PAI-1, were increased in dogs with organ dysfunction secondary to sepsis, compared to healthy dogs. The results of this study broaden our understanding of the pathophysiology of sepsis secondary to endothelial dysfunction. Larger studies are required to investigate clinical utility of these biomarkers for prediction of outcome.

## Data availability statement

The raw data supporting the conclusions of this article will be made available by the authors, without undue reservation.

## Ethics statement

This study was reviewed and approved by the University of Melbourne Animal Ethics Committee and the Murdoch University Animal Ethics Committee. Written informed consent was obtained from the owners for the participation of their animals in this study.

## Author contributions

SG designed and conducted the study (Melbourne site), analyzed and interpreted data, and wrote and critically reviewed the manuscript. LiS designed and conducted the study (Perth site), analyzed and interpreted data, and wrote and critically reviewed the manuscript. MB designed the study, organized the trial, developed the research database, analyzed, and interpreted data, and wrote and critically reviewed the manuscript. AW contributed to study design, data analysis and writing and critically reviewing the manuscript. CS contributed to the study design, data collection and writing and critically reviewing of the manuscript. DH and SB contributed to the study design and writing and critically reviewing of the manuscript. JD and LeS contributed to data collection and analysis and writing and critically reviewing of the manuscript. All authors contributed to the article and approved the submitted version.

## Funding

Funding for this study was provided by an internal grant of the Veterinary School of the University of Melbourne.

## Conflict of interest

The authors declare that the research was conducted in the absence of any commercial or financial relationships that could be construed as a potential conflict of interest.

## Publisher’s note

All claims expressed in this article are solely those of the authors and do not necessarily represent those of their affiliated organizations, or those of the publisher, the editors and the reviewers. Any product that may be evaluated in this article, or claim that may be made by its manufacturer, is not guaranteed or endorsed by the publisher.

## Abbreviations

APPLE_FAST_: Acute patient physiological and laboratory evaluation fast score
aPTT: Activated partial thromboplastin time
CRP: C-reactive protein
HA: Hyaluronan
IL: Interleukin
MCP-1: Monocyte chemoattractant protein-1
PAI-1: Plasminogen activator inhibitor-1
PT: Prothrombin time
VEGF: Vascular endothelial growth factor
vWF: von Willebrand factor

## References

[1] Soo A Zuege DJ Fick GH Niven DJ Berthiaume LR Stelfox HT. Describing organ dysfunction in the intensive care unit: a cohort study of 20,000 patients. Crit Care. (2019) 23:186. doi: 10.1186/s13054-019-2459-9, PMID: 31122276 PMC6533687

[2] Kenney EM Rozanski EA Rush JE De Laforcade-Buress AM Berg JR Silverstein DC. Association between outcome and organ system dysfunction in dogs with sepsis: 114 cases (2003-2007). J Am Vet Med Assoc. (2010) 236:83–7. doi: 10.2460/javma.236.1.83, PMID: 20043806

[3] Summers AM Vezzi N Gravelyn T Culler C Guillaumin J. Clinical features and outcome of septic shock in dogs: 37 cases (2008-2015). J Vet Emerg Crit Care. (2020) 31:360–70. doi: 10.1111/vec.13038, PMID: 33382202

[4] Angus DC Van der Poll T. Severe sepsis and septic shock. N Engl J Med. (2013) 369:840–51. doi: 10.1056/NEJMra1208623, PMID: 23984731

[5] Ince C Mayeux PR Nguyen T Gomez H Kellum JA Ospina-Tascón GA. The endothelium in sepsis. Shock. (2016) 45:259–70. doi: 10.1097/shk.0000000000000473, PMID: 26871664 PMC5281063

[6] Opal SM Van der Poll T. Endothelial barrier dysfunction in septic shock. J Intern Med. (2015) 277:277–93. doi: 10.1111/joim.12331 25418337

[7] Aird WC. Endothelium as a therapeutic target in sepsis. Curr Drug Targets. (2007) 8:501–7. doi: 10.2174/138945007780362782 17430120

[8] Gaudette S Hughes D Boller M. The endothelial glycocalyx: structure and function in health and critical illness. J Vet Emerg Crit Care. (2020) 30:117–34. doi: 10.1111/vec.12925, PMID: 32067360

[9] Xing K Murthy S Liles WC Singh JM. Clinical utility of biomarkers of endothelial activation in sepsis--a systematic review. Crit Care. (2012) 16:R7. doi: 10.1186/cc11145, PMID: 22248019 PMC3396237

[10] Skibsted S Jones AE Puskarich MA Arnold R Sherwin R Trzeciak S. Biomarkers of endothelial cell activation in early sepsis. Shock. (2013) 39:427–32. doi: 10.1097/SHK.0b013e3182903f0d, PMID: 23524845 PMC3670087

[11] Yano K Liaw PC Mullington JM Shih SC Okada H Bodyak N. Vascular endothelial growth factor is an important determinant of sepsis morbidity and mortality. J Exp Med. (2006) 203:1447–58. doi: 10.1084/jem.20060375, PMID: 16702604 PMC2118329

[12] Shapiro NI Schuetz P Yano K Sorasaki M Parikh SM Jones AE. The association of endothelial cell signaling, severity of illness, and organ dysfunction in sepsis. Crit Care. (2010) 14:R182. doi: 10.1186/cc9290, PMID: 20942957 PMC3219288

[13] Vassiliou AG Mastora Z Orfanos SE Jahaj E Maniatis NA Koutsoukou A. Elevated biomarkers of endothelial dysfunction/activation at ICU admission are associated with sepsis development. Cytokine. (2014) 69:240–7. doi: 10.1016/j.cyto.2014.06.010, PMID: 25016133

[14] Ostrowski SR Haase N Müller RB Møller MH Pott FC Perner A. Association between biomarkers of endothelial injury and hypocoagulability in patients with severe sepsis: a prospective study. Crit Care. (2015) 19:191. doi: 10.1186/s13054-015-0918-5, PMID: 25907781 PMC4423170

[15] Dellinger RP Levy MM Rhodes A Annane D Gerlach H Opal SM. Surviving sepsis campaign: international guidelines for management of severe sepsis and septic shock: 2012. Crit Care Med. (2013) 41:580–637. doi: 10.1097/CCM.0b013e31827e83af 23353941

[16] Hayes G Mathews K Doig G Kruth S Boston S Nykamp S. The acute patient physiologic and laboratory evaluation (APPLE) score: a severity of illness stratification system for hospitalized dogs. J Vet Int Med. (2010) 24:1034–47. doi: 10.1111/j.1939-1676.2010.0552.x, PMID: 20629945

[17] Harris PA Taylor R Thielke R Payne J Gonzalez N Conde JG. Research electronic data capture (REDCap)--a metadata-driven methodology and workflow process for providing translational research informatics support. J Biomed Inform. (2009) 42:377–81. doi: 10.1016/j.jbi.2008.08.010, PMID: 18929686 PMC2700030

[18] Stokol T. A study of von Willebrand disease in dogs in Australia. Veterinary Science Theses: University of Melbourne (1993).

[19] Stokol T Parry B. Stability of canine factor VIII and von Willebrand factor antigen concentration in the frozen state. Res Vet Sci. (1995) 59:156–9. doi: 10.1016/0034-5288(95)90051-9, PMID: 8525106

[20] R Core Team. R: A language and environment for statistical computing. R foundation for statistical computing. Vienna, Austria: R Core Team (2021).

[21] van de Schoot R Kaplan D Denisson J Asendorpf JB van Aken MAG. A gentle introduction to Bayesian analysis: applications to developmental research. Child Dev. (2013) 85:842–60. doi: 10.1111/cdev.12169, PMID: 24116396 PMC4158865

[22] Kruschke JK. Bayesian estimation supersedes the t test. J Exp Psychol Gen. (2013) 142:573–603. doi: 10.1037/a0029146, PMID: 22774788

[23] Bürkner P-C. Brms: an R package for Bayesian multilevel models using Stan. J Stat Softw. (2017) 80:1–28. doi: 10.18637/jss.v080.i01

[24] Steppan J Hofer S Funke B Brenner T Henrich M Martin E. Sepsis and major abdominal surgery lead to flaking of the endothelial glycocalix. J Surg Res. (2011) 165:136–41. doi: 10.1016/j.jss.2009.04.034, PMID: 19560161

[25] Ostrowski SR Pedersen SH Jensen JS Mogelvang R Johansson PI. Acute myocardial infarction is associated with endothelial glycocalyx and cell damage and a parallel increase in circulating catecholamines. Crit Care. (2013) 17:R32. doi: 10.1186/cc12532, PMID: 23433357 PMC4057225

[26] Anand D Ray S Srivastava LM Bhargava S. Evolution of serum hyaluronan and syndecan levels in prognosis of sepsis patients. Clin Biochem. (2016) 49:768–76. doi: 10.1016/j.clinbiochem.2016.02.014, PMID: 26953518

[27] Mittermayer F Pleiner J Schaller G Weltermann A Kapiotis S Jilma B. Marked increase in vascular endothelial growth factor concentrations during *Escherichia coli* endotoxin-induced acute inflammation in humans. Eur J Clin Invest. (2003) 33:758–61. doi: 10.1046/j.1365-2362.2003.01192.x, PMID: 12925034

[28] van Der Flier M Coenjaerts F Kimpen JL Hoepelman AM Geelen SP. *Streptococcus pneumoniae* induces secretion of vascular endothelial growth factor by human neutrophils. Infect Immun. (2000) 68:4792–4. doi: 10.1128/iai.68.8.4792-4794.2000, PMID: 10899891 PMC98440

[29] McCourt M Wang JH Sookhai S Redmond HP. Proinflammatory mediators stimulate neutrophil-directed angiogenesis. Arch Surg. 134:1325:1331. doi: 10.1001/archsurg.134.12.1325 10593330

[30] Jeong SJ Han SH Kim CO Choi JY Kim JM. Anti-vascular endothelial growth factor antibody attenuates inflammation and decreases mortality in an experimental model of severe sepsis. Crit Care. (2013) 17:R97. doi: 10.1186/cc12742, PMID: 23710641 PMC4056034

[31] Ferrara N Gerber HP LeCouter J. The biology of VEGF and its receptors. Nat Med. (2003) 9:669–76. doi: 10.1038/nm0603-669 12778165

[32] Reinders ME Sho M Izawa A Wang P Mukhopadhyay D Koss KE. Proinflammatory functions of vascular endothelial growth factor in alloimmunity. J Clin Invest. (2003) 112:1655–65. doi: 10.1172/jci17712, PMID: 14660742 PMC281640

[33] van der Flier M van Leeuwen HJ van Kessel KP Kimpen JL Hoepelman AI Geelen SP. Plasma vascular endothelial growth factor in severe sepsis. Shock. (2005) 23:35–8. doi: 10.1097/01.shk.0000150728.91155.41 15614129

[34] Karlsson S Pettilä V Tenhunen J Lund V Hovilehto S Ruokonen E. Vascular endothelial growth factor in severe sepsis and septic shock. Anesth Analg. (2008) 106:1820–6. doi: 10.1213/ane.0b013e31816a643f 18499616

[35] Hou PC Filbin MR Wang H Ngo L Huang DT Aird WC. Endothelial permeability and hemostasis in septic shock: results from the ProCESS trial. Chest. (2017) 152:22–31. doi: 10.1016/j.chest.2017.01.010, PMID: 28109962 PMC5577354

[36] Silverstein DC Montealegre C Shofer FS Otto CM. The association between vascular endothelial growth factor levels and clinically evident peripheral edema in dogs with systemic inflammatory response syndrome. J Vet Emerg Crit Care. (2009) 19:459–66. doi: 10.1111/j.1476-4431.2009.00457.x, PMID: 19821887

[37] König M Nentwig A Marti E Mirkovitch J Adamik KN Schuller S. Evaluation of plasma angiopoietin-2 and vascular endothelial growth factor in healthy dogs and dogs with systemic inflammatory response syndrome or sepsis. J Vet Intern Med. (2019) 33:569–77. doi: 10.1111/jvim.15369, PMID: 30575998 PMC6430886

[38] Sonderegger F Nentwig A Schweighauser A Francey T Marti E Mirkovitch J. Association of markers of endothelial activation and dysfunction with occurrence and outcome of pulmonary hemorrhage in dogs with leptospirosis. J Vet Intern Med. (2021 Jul) 35:1789–99. doi: 10.1111/jvim.16163, PMID: 34076314 PMC8295707

[39] Marechal X Favory R Joulin O Montaigne D Hassoun S Decoster B. Endothelial glycocalyx damage during endotoxemia coincides with microcirculatory dysfunction and vascular oxidative stress. Shock. (2008) 29:572–6. doi: 10.1097/SHK.0b013e318157e926, PMID: 18414231

[40] Iba T Levy JH Aihara K Kadota K Tanaka H Sato K. Newly developed recombinant antithrombin protects the endothelial glycocalyx in an endotoxin-induced rat model of sepsis. Int J Mol Sci. (2020) 22:176. doi: 10.3390/ijms22010176 33375342 PMC7795760

[41] Byrne L Obonyo NG Diab SD Dunster KR Passmore MR Boon AC. Unintended consequences: fluid resuscitation worsens shock in an ovine model of endotoxemia. Am J Resp Crit Care Med. (2018) 198:1043–54. doi: 10.1164/rccm.201801-0064OC, PMID: 29882682 PMC7613331

[42] Yagmur E Koch A Haumann M Kramann R Trautwein C Tacke F. Hyaluronan serum concentrations are elevated in critically ill patients and associated with disease severity. Clin Biochem. (2012) 45:82–7. doi: 10.1016/j.clinbiochem.2011.10.016, PMID: 22085533

[43] Sallisalmi M Tenhunen J Kultti A Tammi M Pettila V. Plasma hyaluronan and hemorheology in patients with septic shock: a clinical and experimental study. Clin Hemorheol Microcirc. (2014) 56:133–44. doi: 10.3233/ch-131677, PMID: 23380965

[44] Smart L Macdonald SPJ Burrows S Bosio E Arendts G Fatovich DM. Endothelial glcyocalyx biomarkers increase in patients with infection during emergency department treatment. J Crit Care. (2017) 42:304–9. doi: 10.1016/j.jcrc.2017.07.001 28822340

[45] Smart L Bosio E Macdonald SPJ Dull R Fatovich DM Neil C. Glycocalyx biomarker syndecan-1 is a stronger predictor of respiratory failure in patients with sepsis due to pneumonia, compared to endocan. J Crit Care. (2018) 47:93–8. doi: 10.1016/j.jcrc.2018.06.015, PMID: 29936329

[46] Shaw K Bersenas A Bateman S Blois S Guieu LV Wood R. Use of serum hyaluronic acid as a biomarker of endothelial glycocalyx degradation in dogs with septic peritonitis. Am J Vet Res. (2021) 82:566–73. doi: 10.2460/ajvr.82.7.566, PMID: 34166092

[47] Smart L Hughes D. The effects of resuscuitative fluid therapy on the endothelial surface layer. Front Vet Sci. (2021) 8:661660. doi: 10.3389/fvets.2021.661660, PMID: 34026896 PMC8137965

[48] Hoshino K Kitamura T Nakamura Y Irie Y Matsumoto N Kawano Y. Usefulness of plasminogen activator inhibitor-1 as a predictive marker of mortality in sepsis. J Intensive Care. (2017) 5:42. doi: 10.1186/s40560-017-0238-8, PMID: 28702197 PMC5504563

[49] Koyama K Madoiwa S Nunomiya S Koinuma T Wada M Sakata A. Combination of thrombin-antithrombin complex, plasminogen activator inhibitor-1, and protein C activity for early identification of severe coagulopathy in initial phase of sepsis: a prospective observational study. Crit Care. (2014) 18:R13. doi: 10.1186/cc13190, PMID: 24410881 PMC4056264

[50] Hoshino K Nakashio M Maruyama J Irie Y Kawano Y Ishikura H. Validating plasminogen activator inhibitor-1 as a poor prognostic factor in sepsis. Acute Med Surg. (2020) 7:e581. doi: 10.1002/ams2.581, PMID: 33173586 PMC7642588

[51] Wiinberg B Jensen AL Johansson PI Kjelgaard-Hansen M Rozanski E Tranholm M. Development of a model based scoring system for diagnosis of canine disseminated intravascular coagulation with independent assessment of sensitivity and specificity. Vet J. (2010) 185:292–8. doi: 10.1016/j.tvjl.2009.06.003, PMID: 19586785

[52] Goggs R Mastrocco A Brooks MB. Retrospective evaluation of 4 methods for outcome prediction in overt disseminated intravascular coagulation in dogs (2009-2014): 804 cases. J Vet Emerg Crit Care. (2018) 28:541–50. doi: 10.1111/vec.12777, PMID: 30302935

[53] Bernardo A Ball C Nolasco L Moake JF Dong JF. Effects of inflammatory cytokines on the release and cleavage of the endothelial cell-derived ultralarge von Willebrand factor multimers under flow. Blood. (2004) 104:100–6. doi: 10.1182/blood-2004-01-0107, PMID: 15026315

[54] Kremer Hovinga JA Zeerleder S Kessler P De Wit TR Van Mourik JA Hack CE. ADAMTS-13, von Willebrand factor and related parameters in severe sepsis and septic shock. J Thromb Haemost. (2007) 5:2284–90. doi: 10.1111/j.1538-7836.2007.02743.x, PMID: 17764538

[55] Rubin DB Wiener-Kronish JP Murray JF Green DR Turner J Luce JM. Elevated von Willebrand factor antigen is an early plasma predictor of acute lung injury in nonpulmonary sepsis syndrome. J Clin Invest. (1990) 86:474–80. doi: 10.1172/jci114733, PMID: 2384595 PMC296749

[56] Novotny MJ Turrentine MA Johnson GS Adams HR. Experimental endotoxemia increases plasma von Willebrand factor antigen concentrations in dogs with and without free-radical scavenger therapy. Circ Shock. (1987) 23:205–13. PMID: 3322605

[57] Rogers CL Rozanski EA. Von Willebrand factor antigen concentration in dogs with sepsis. J Vet Intern Med. (2010) 24:229–30. doi: 10.1111/j.1939-1676.2009.0436.x, PMID: 20002549

[58] Kang S Tanaka T Inoue H Ono C Hashimoto S Kioi Y. IL-6 trans-signaling induces plasminogen activator inhibitor-1 from vascular endothelial cells in cytokine release syndrome. Proc Natl Acad Sci U S A. (2020) 117:22351–6. doi: 10.1073/pnas.2010229117, PMID: 32826331 PMC7486751

[59] Goggs R Letendre JA. Evaluation of the host cytokine response in dogs with sepsis and noninfectious systemic inflammatory response syndrome. J Vet Emerg Crit Care. (2019) 29:593–603. doi: 10.1111/vec.12903, PMID: 31637812

[60] Johnson V Burgess B Morley P Bragg R Avery A Dow S. Comparison of cytokine responses between dogs with sepsis and dogs with immune-mediated hemolytic anemia. Vet Immunol Immunopathol. (2016) 180:15–20. doi: 10.1016/j.vetimm.2016.08.010, PMID: 27692090

[61] Lippi G Plebani M Favaloro EJ. Interference in coagulation testing: focus on spurious hemolysis, icterus, and lipemia. Sem Thrombos Hemostas. (2013) 39:258–66. doi: 10.1055/s-0032-1328972, PMID: 23229354

[62] Moser J Meyers KM Meinkoth JH Brassard JA. Temporal variation and factors affecting measurement of canine von Willebrand factor. Am J Vet Res. (1996) 57:1288–93. PMID: 8874720

[63] Woodhams B Girardot O Blanco MJ Colesse G Gourmelin Y. Stability of coagulation proteins in frozen plasma. Blood Coagul Fibrinolysis. (2001) 12:229–36. doi: 10.1097/00001721-200106000-00002 11460005

